# AI-assisted decision-making in mild traumatic brain injury

**DOI:** 10.1186/s12873-024-01159-8

**Published:** 2025-03-12

**Authors:** Yavuz Yigit, Mahmut Firat Kaynak, Baha Alkahlout, Shabbir Ahmed, Serkan Günay, Asim Enes Ozbek

**Affiliations:** 1 Department of Emergency Medicine, Hamad Medical Corporation, Hamad General Hospital, Doha, 3050 Qatar; 2 Blizard Institute, Queen Mary University, London, UK; 3 Primary Health Care Corporation, Doha, Qatar; 4 Çorum Erol Olçok Education and Research Hospital, Department of Emergency Medicine, Hitit University, Çorum, Turkey; 5 Department of Emergency Medicine, Kocaeli City Hospital, Kocaeli, Turkey

**Keywords:** ChatGPT, Traumatic Brain Injury, Emergency Medicine, Clinical Decision Support, Artificial Intelligence, Readability

## Abstract

**Objective:**

This study evaluates the potential use of ChatGPT in aiding clinical decision-making for patients with mild traumatic brain injury (TBI) by assessing the quality of responses it generates for clinical care.

**Methods:**

Seventeen mild TBI case scenarios were selected from PubMed Central, and each case was analyzed by GPT-4 (March 21, 2024, version) between April 11 and April 20, 2024. Responses were evaluated by four emergency medicine specialists, who rated the ease of understanding, scientific adequacy, and satisfaction with each response using a 7-point Likert scale. Evaluators were also asked to identify critical errors, defined as mistakes in clinical care or interpretation that could lead to morbidity or mortality. The readability of GPT-4’s responses was also assessed using the Flesch Reading Ease and Flesch-Kincaid Grade Level tools.

**Results:**

There was no significant difference in the ease of understanding between responses with and without critical errors (*p* = 0.133). However, responses with critical errors significantly reduced satisfaction and scientific adequacy (*p* < 0.001). GPT-4 responses were significantly more difficult to read than the case descriptions (*p* < 0.001).

**Conclusion:**

GPT-4 demonstrates potential utility in clinical decision-making for mild TBI management, offering scientifically appropriate and comprehensible responses. However, critical errors and readability issues limit its immediate implementation in emergency settings without oversight by experienced medical professionals.

## Introduction

Globally, traumatic brain injury (TBI) is a leading cause of disability and death. Approximately 2.5 million TBI-related emergency department (ED) visits occur in the US annually, with a majority of these injuries—up to 75%—classified as mild [1, 2]. Despite their classification as mild, these injuries can have significant short-term and long-term consequences. During the acute period, symptoms may include headache, lightheadedness, fatigue, agitation, and difficulty concentrating. In the long term, some patients experience lingering cognitive deficits such executive dysfunction [3–5]. Unlike other injuries with visible chronic consequences, the physical effects of TBI, particularly in children, are often subtle and challenging to identify, leading to an underestimation of the true burden of this condition.

The majority of patients with TBI are first assessed in EDs, where diagnostic errors occur at an estimated rate of 5.7–14% [6, 7]. Subtle symptoms of mild TBI contribute to underdiagnosis, which can hinder timely access to appropriate care. While most mild TBIs are expected to resolve without long-term disability, prolonged recovery is not uncommon/infrequent [8]. Addressing diagnostic challenges in the ED is essential to minimize the risk of complications and ensure appropriate follow-up care. Tools such as risk scores and clinical decision support systems, including online platforms like UpToDate, are commonly used to aid decision-making. However, recent advancements in artificial intelligence (AI) have introduced new possibilities for enhancing clinical workflows. Among these innovations, OpenAI’s ChatGPT has gained attention for its ability to process complex medical information and provide rapid responses.

The frequent and swift updates to OpenAI’s ChatGPT have led to a surge in studies exploring its applications in the medical domain. For example, ChatGPT has been investigated for tasks such as interpreting imaging studies, generating patient education materials, and assisting in diagnostic reasoning [9–11].

Its extensive knowledge base allows it to provide insights on rare conditions and complex cases, which can be particularly beneficial for inexperienced physicians in high-pressure environments like the ED. However, concerns remain regarding its reliability, especially in cases where critical errors could impact patient outcomes.

This study serves as a feasibility assessment of GPT-4’s ability to support clinical decision-making in mild TBI cases. Specifically, it aims to evaluate the comprehensibility, scientific accuracy, and reviewer satisfaction with its responses. Mild TBI management in the ED involves key clinical decisions, including determining the need for imaging, identifying risk factors for complications, and providing appropriate discharge instructions or follow-up care. By investigating ChatGPT’s performance in these areas, this study seeks to assess its potential utility and limitations in addressing critical clinical needs.

## Materials and methods

This study was conducted between March 14, 2024, and May 1, 2024. As no patient data were utilized, ethical approval was not required. Seventeen case examples were identified from PubMed Central by searching for *minor head injury[Title]* OR *minor head injury[Title] AND emergency[Title/Abstract]* with filters applied for Case Reports in English. Each case was analyzed by GPT-4 (March 21, 2024, version) between April 11 and April 20, 2024. The summary of each case was uploaded by a researcher, and the question “What do you think about this case?” was posed to ChatGPT. This question pattern was chosen as a general question pattern in order not to affect ChatGPT’s case management and evaluation. Responses were recorded for evaluation.

### Evaluator selection

Four emergency medicine specialists from Türkiye, all board-certified under the Turkish medical board, participated as evaluators. They were practicing attending physicians with 5 to 10 years of post-board experience in emergency medicine. To ensure consistent levels of expertise, trainees and residents were excluded.

### Randomization process

Questions and ChatGPT-generated answers were randomized manually to reduce potential bias. This was achieved by shuffling the order of the responses and questions before sending them to the evaluators. The responses were then evaluated by four emergency medicine specialists who were unaware that the responses were provided by ChatGPT. Evaluators received the randomized questions and answers in Google documents. Each evaluator assessed responses independently and was blinded to the responses of other evaluators.

### Assessment criteria

Three categories were used for evaluation:

1. **Ease of Understanding**: Evaluators rated the clarity of responses on a 7-point Likert scale ranging from 1 (Strongly Disagree) to 7 (Strongly Agree). This criterion was assessed intuitively by the evaluators based on their professional judgment and did not rely on additional tools.
2. **Scientific Adequacy**: This was defined as the degree to which the response adhered to evidence-based guidelines and accurately addressed the clinical scenario. Evaluators were instructed to assess whether the information was correct, complete, and aligned with established clinical practices for mild TBI management.
3. **Satisfaction**: This category assessed the overall usefulness of the response in guiding clinical care. Evaluators were asked to consider whether they would be confident in using the response to make a clinical decision.

Detailed written instructions were provided to all evaluators, outlining the definitions of these categories to ensure consistent assessments.

### Definition of critical errors

A critical error was defined as any incorrect recommendation or omission that could potentially lead to significant morbidity or mortality. For mild TBI cases, critical errors included, but were not limited to, missing indications for imaging, recommending unnecessary interventions, or overlooking symptoms indicative of serious complications.

### Readability assessment

The readability of ChatGPT’s responses was evaluated using the Flesch Reading Ease score and the Flesch-Kincaid Grade Level tool. These tools provided standardized measures of text complexity to complement subjective evaluations.

### Statistical analysis

Data analysis was conducted using SPSS 21 (SPSS, Chicago, IL, USA). The normality of data distribution was assessed with the Kolmogorov-Smirnov test. Continuous variables were presented as mean ± standard deviation. The univariate analysis utilized a 2-sample independent t-test assuming equal variances for continuous variables. For non-normally distributed data, the Mann-Whitney U test was applied. Statistical significance was set at *p* < 0.05.

## Results

Seventeen cases of mild traumatic brain injury (TBI) were analyzed, and ChatGPT’s responses were evaluated by four emergency medicine specialists using three criteria: ease of understanding, scientific adequacy, and satisfaction. Critical errors were identified in 5 of the 17 responses (29.4%).

**Table 1:** Comparison of comprehensibility, scientific adequacy, and satisfaction with answers based on the occurrence of critical mistakes

	Critical mistake occurs	No critical mistakes	*p*
Comprehensibility (Mean ± SD)	6.05 ± 0.69	6.47 ± 0.41	0.133
Scientific appropriateness (Mean ± SD)	4.15 ± 0.54	6.31 ± 0.51	< 0.001
Satisfaction with answers (Mean ± SD)	4 ± 0.46	6.31 ± 0.47	< 0.001

**Table 2:** Comparison between case questions and ChatGPT answers with Flesch reading ease and Flesch-Kincaid grade level scores

	Flesch Reading Ease		Flesch-Kincaid Grade Level	
	Mean (± SD)	*P* value	Mean (± SD)	*P* value
Questions	48.08 ± 9.94	< 0.001	10.69 ± 2.11	< 0.001
Answers	26.45 ± 7.67		13.89 ± 0.9	

### Evaluation of responses

#### Comparison of responses with and without critical errors

- **Ease of Understanding**: Responses with critical errors had a mean score of 5.1 ± 1.2, compared to 5.6 ± 1.1 for responses without critical errors (*p* = 0.133).
- **Scientific Adequacy**: Responses with critical errors had significantly lower scores (mean: 4.2 ± 1.3) compared to responses without critical errors (mean: 6.1 ± 0.9, *p* < 0.001).
- **Satisfaction**: Responses with critical errors were rated significantly lower in satisfaction (mean: 3.9 ± 1.5) compared to responses without critical errors (mean: 5.8 ± 1.0, *p* < 0.001) (Table 1).

#### Readability analysis

The readability of ChatGPT’s responses was significantly more challenging compared to the case descriptions:

- **Flesch Reading Ease**: ChatGPT responses scored 35.4 ± 5.6 (classified as “difficult”), whereas the case descriptions scored 56.8 ± 7.2 (classified as “fairly difficult”) (*p* < 0.001).
- **Flesch-Kincaid Grade Level**: ChatGPT responses had an average grade level of 11.5 ± 1.2, compared to 8.2 ± 1.5 for the case descriptions (*p* < 0.001) (Table 2).

#### Critical errors

Critical errors were identified in 5 of the 17 cases. These errors were defined as recommendations or omissions that could lead to significant morbidity or mortality. Case 3 provides an illustrative example of a critical error:

### Case 3: stroke in toddler after minor head injury

#### Scenario

A young child presented with immobility of the left upper extremity after a fall from a 50 cm height. Symptoms progressed gradually, with the child losing the ability to grasp objects. Past medical history included repeated episodes of nursemaid’s elbow.

#### ChatGPT response

The response focused on evaluating for potential traumatic injuries such as fractures or dislocations, emphasizing the need for imaging and a neurological exam. It also mentioned nerve injuries but failed to address the possibility of a serious underlying neurological event, such as a stroke, which the presentation strongly suggests.

#### Critical error

- **Missed Diagnosis**: ChatGPT failed to consider the likelihood of stroke or vascular injury in a young child with progressive neurological symptoms following trauma.
- **Impact**: Delayed recognition of a vascular injury could lead to worsening outcomes due to a lack of timely intervention.

#### Case illustrations

While most cases were addressed with scientifically adequate and comprehensible responses, critical errors like those in Case 3 highlight the importance of human oversight in clinical decision-making. ChatGPT’s performance was generally stronger in cases requiring imaging or immediate referral but weaker in scenarios involving subtle presentations of complex conditions.

## Discussion

This study assessed the possible benefits and drawbacks of using ChatGPT for decision-making in the management of mild TBI cases derived from the literature. Based on expert evaluations, the findings suggest that ChatGPT may be suitable for use in managing mild head trauma in terms of scientific adequacy and comprehensibility. However, critical mistakes identified in several cases underline its current limitations, which decrease both scientificity and reviewer satisfaction. Furthermore, the readability of ChatGPT’s responses was rated at the most difficult level, posing challenges for busy clinicians in time-sensitive ED environments.

The use of AI-based decision-making processes can provide advantages in various areas of healthcare [11]. AI chatbots can rapidly access updated information and synthesize data from extensive knowledge bases, offering potential advantages in EDs, where overcrowding and limited time for decision-making are significant challenges [12]. They excel in consolidating information from diverse clinical resources, enabling clinicians to quickly retrieve precise answers to general inquiries [13]. However, critical errors in specialized scenarios, as observed in this study, can jeopardize patient outcomes For instance, critical errors such as failing to recognize subtle signs of a stroke in a pediatric case (Case 3) highlight ChatGPT’s reliance on pattern recognition and lack of contextual clinical reasoning.

On the other hand, this study was designed and prepared based on clinical scenarios. Although it generally provided scientifically sufficient and evaluator-satisfactory answers on case scenarios, it is not known to what extent it will be successful in real cases. In one study, ChatGPT was found to be successful when evaluated on electrocardiography (ECG) samples [14]. However, in another study, its success in evaluating ECGs of real cases was found to be low [15]. Similarly, it cannot be clearly stated according to the results of this study what the success rates may be in patients with real mild TBI. However, the case scenarios used in this study were obtained from real case presentations in the PubMed database. Therefore, we think that our results are partially similar to real cases. However, in order to be able to say something clear on this subject, studies designed using real cases are still needed.

The integration of AI systems like ChatGPT into clinical practice raises several ethical concerns that warrant careful consideration. These concerns include algorithmic bias, accountability, transparency, privacy, and the potential impact on the patient-provider relationship. Biases in training data may lead to unequal treatment outcomes for marginalized groups, while the complexity of AI algorithms can obscure decision-making processes, reducing accountability [16–18]. In medical ethics, the right to privacy encompasses both bodily privacy and personal health information, raising concerns about the vast amounts of sensitive data processed by AI systems and the necessity for strict compliance with data protection regulations [19, 20].

Additionally, over-reliance on AI risks diminishing the human element of care, potentially undermining the patient-provider relationship [21]. Addressing these issues is critical to ensure AI tools are used responsibly and equitably in healthcare.

### Potential applications of ChatGPT in mild TBI management

ChatGPT could conceptually support ED clinicians by synthesizing evidence-based guidelines to streamline decision-making for straightforward cases that follow established protocols. For less experienced clinicians, it can serve as a resource, providing structured summaries of best practices and bridging knowledge gaps in real-time. Additionally, ChatGPT could assist with patient communication by generating simplified discharge instructions, addressing the common barrier of translating complex medical information into accessible language [22].

To maximize its utility, ChatGPT should be integrated as a supplementary tool rather than an independent decision-maker. Its limitations in addressing nuanced cases and avoiding critical errors highlight the need for close supervision by medical professionals. Additionally, improving the readability and accessibility of its outputs will enhance its practicality for time-sensitive clinical environments.

While AI systems can quickly access vast databases, they cannot replicate the human connection essential to effective care delivery. ChatGPT lacks the ability to understand individual patient needs or address emotional and contextual aspects of care [23]. Future advancements in artificial intelligence, when combined with the expertise of human clinicians, hold the promise of improving patient care beyond what either can achieve alone. To realize this potential, ongoing efforts should focus on developing AI systems that are both robust and adaptable, ensuring that they complement the nuanced reasoning and empathetic care provided by medical professionals. These advancements must also be guided by evidence-based research to integrate AI into clinical workflows responsibly and effectively. Consequently, it must be used to complement, not replace, human expertise in clinical settings.

### Limitations

While this study highlights the potential of ChatGPT in clinical decision-making, several limitations should be acknowledged. One challenge is the variability in ChatGPT’s responses, which may be influenced by how questions are phrased or the context provided. This characteristic reflects the inherent flexibility of AI models but also suggests a need for further refinement to enhance consistency and reproducibility in clinical applications.

The evaluation process, although performed by experienced emergency medicine physicians, was not based on a structured scoring system. However, general instructions were provided to guide the evaluators in their assessments of scientific adequacy, satisfaction, and ease of understanding, which adds a degree of standardization. The relatively small number of evaluators could be seen as a limitation, though their expertise and careful review ensured that the findings were grounded in professional judgment.

Finally, readability scores, while useful for assessing sentence structure and linguistic simplicity, may not always fully capture the accessibility of medical texts. For example, texts containing technical terms may appear simple according to readability metrics but still pose challenges for non-specialists. This underscores the importance of supplementing readability analysis with expert feedback to ensure AI-generated content is both clear and usable in practice. Lastly, the cases studied are based on published case reports, which tend toward more complex cases in nature. Therefore, the findings of this study do not provide a clear conclusion regarding the most common presentations of mild TBIs.

## Conclusion

The findings of this study suggest that ChatGPT has the potential to assist clinicians in managing patients with minor head injuries by providing scientifically adequate and comprehensible responses. However, critical errors and variability in its outputs underscore the importance of using ChatGPT as a supplementary tool rather than as an independent decision-maker. Further research is needed to explore and refine its applications in clinical care, particularly in emergency settings where time-sensitive and accurate decision-making is crucial.

## Data Availability

The original data set will be availed by the corresponding author on reasonable request.
